# Anti-inflammatory Property of Galectin-1 in a Murine Model of Allergic Airway Inflammation

**DOI:** 10.1155/2019/9705327

**Published:** 2019-05-12

**Authors:** Yunxiang Lv, Mengyuan Dai, Muzi Wang, Fangfang Chen, Rongyu Liu

**Affiliations:** Department of Pulmonary Medicine, Anhui Geriatric Institute, The First Affiliated Hospital of Anhui Medical University, Jixi Road 218, Hefei, Anhui 230022, China

## Abstract

Galectin-1 (Gal-1) has immunomodulatory activities in various allergic inflammatory disorders, but its potential anti-inflammatory properties on allergic airway diseases have not been confirmed. We explored the pharmacological effects of Gal-1 on the progression of allergic airway inflammation and investigated the underlying mechanism. Female C57BL/6 mice were sensitized on day 0 and challenged with ovalbumin (OVA) on days 14-17 to establish an allergic airway inflammation model. In the challenge phase, a subset of mice was treated intraperitoneally with recombinant Gal-1 (rGal-1) or dexamethasone (Dex). We found that rGal-1 inhibited pulmonary inflammatory cell recruitment, mucus secretion, bronchoalveolar lavage fluid (BALF) inflammatory cell infiltration, and cytokine production. The treatment also suppressed the infiltration of eosinophils into the allergic lung as indicated by decreased expression levels of eotaxin and eosinophil peroxidase (EPX). However, only the expression levels of IL-25, neither IL-33 nor TSLP, were significantly decreased in the lung by rGal-1 treatment. These immunomodulatory effects in the allergic lung were correlated with the activation of extracellular signal-regulated kinase (ERK) signaling pathway and downregulation of endogenous Gal-1. In addition, rGal-1 reduced the plasma concentrations of anti-OVA immunoglobulin E (IgE) and IL-17. Our findings suggest that rGal-1 is an effective therapy for allergic airway inflammation in a murine model and may be a potential pharmacological target for allergic airway inflammatory diseases.

## 1. Introduction

The universally acknowledged pathogenesis of allergic airway disease is attributed to imbalances in the adaptive immune system, including dysfunction of T helper (Th) cells and elevated production of IgE, which result in type 2 cytokine production and inflammatory cells infiltrating the airways [1–3]. Currently, inhaled or systemic glucocorticoids are the most effective and widely applied therapy for allergic airway inflammation. Unfortunately, approximately 10% of patients do not respond to maximal, optimally delivered therapy, resulting in increased morbidity and higher costs [4–6]. Therefore, it is important to identify potential new therapeutic targets for allergic airway disease.

Galectin-1 (Gal-1) is a glycan-binding protein that has a broad distribution in adult tissues, including the lungs, and is expressed by various cell types, such as macrophages, dendritic cells, activated T cells, stromal cells, endothelial cells, and epithelial cells [7, 8]. Increasing evidence from multiple inflammatory disease models supports the critical role of exogenous and endogenous Gal-1 in resolving inflammation [9, 10]. In an IgE-mediated allergic conjunctivitis model, administration of recombinant Gal-1 (rGal-1) resulted in the resolution of clinical signs of conjunctivitis and decreased the production of Th2 cytokines and chemokines [11]. Similarly, Gal-1 pretreatment yielded reduced intestinal allergic inflammation by restoring IL-10 expression of the intestine in a model of oral-intestinal allergy syndrome [12]. Moreover, in an atopic dermatitis model, rGal-1 decreased the clinical signs, diminished local eotaxin levels, and suppressed the infiltration of eosinophils and mast cells by activation of extracellular signal-regulated kinase (ERK) [13]. Gal-1 may work either intracellularly or extracellularly. The protein functions to resolve acute and chronic inflammation by affecting processes, such as immune cell adhesion, migration, activation, proliferation, differentiation, apoptosis, and signaling [9, 10, 14]. Additionally, Gal-1 has displayed anti-inflammatory effects in models of acute inflammation in which neutrophil recruitment and mast cell degranulation were inhibited [15, 16]. The immunosuppressive role of Gal-1 was further supported by the finding that T cells (Tregs) from Gal-1 null mice showed reduced regulatory activity [17]. Furthermore, macrophages and cells of the microglia compartment exhibited a shift toward the M2 phenotype and decreased production of proinflammatory cytokines upon exposure to this lectin [18].

Little is known concerning the potential role of Gal-1 in allergic airway inflammation. By far, the most studied and well-established role of Gal-1 that may be correlated with allergic inflammation is the maintenance of T cell homeostasis through the lectin's ability to induce apoptosis of activated T cells and thus mediate a strong ongoing immune response [8, 19]. Other known Gal-1 effects that could potentially have a beneficial effect during asthma include induction of IL-10 production by T cells [20, 21], supporting the inhibitory function of regulatory T cells (Tregs) [17], and suppression of inflammatory cytokine release by T cells [22]. Compared to wild-type mice, Gal-1-deficient mice challenged with ovalbumin (OVA) exhibited increased recruitment of eosinophils in the airways, had an increased propensity to develop airway hyperresponsiveness, and showed significantly elevated levels of TNF-*α* in lung tissue [23].

In the present study, we hypothesized that Gal-1 might be able to mediate allergic airway inflammation. We thus evaluated the effects of Gal-1 administration and investigated the mechanism in a mouse model of ovalbumin-induced allergic airway inflammation.

## 2. Material and Methods

### 2.1. Animals

Female C57BL/6 mice (6-8 weeks, 20-25 g) were obtained from the Shanghai Laboratory Animal Center and were housed under specific pathogen-free conditions at the Department of Laboratory Animals Center. All of the animal experiments were strictly conducted under protocols approved by the Institutional Animal Care and Use Committee of the University of Science and Technology of China.

### 2.2. Experimental Protocols and Treatments

The protocol applied to establish OVA-induced allergic airway disease in mice was as described in a previous study [24]. Briefly, mice were randomly distributed into four groups (*n* = 6 each): control (Con), OVA, rGal-1, and Dex. The mice were sensitized by intraperitoneal injection of 10 *μ*g OVA (Chicken Egg OVA, Grade V; Sigma) and 1 mg aluminum potassium sulfate (Sangon Biotech, Shanghai, China) in 0.5 ml saline on day 0, then challenged for 30 min per day with 1% aerosolized OVA on days 14–17. Control mice were saline-sensitized and challenged with nebulized saline solution. On days 14–17, rGal-1 and Dex mice were pretreated with recombinant Gal-1 protein (3 *μ*g/animal, Peprotech EC Ltd., London, UK) or dexamethasone (1 mg/kg, Sigma-Aldrich) diluted in 0.1 ml of sterile saline 30 min prior the OVA challenge. The doses of rGal-1 and Dex were described previously [11, 13]. All of the mice were killed 24 hours after the final challenge. The left lung was harvested and subjected to bronchoalveolar lavage and subsequent differential cell counting and ELISA analysis, and the right lung was used for histopathological analysis and further Western blotting assays.

### 2.3. Histological Analysis

Lung tissues were fixed in 4% paraformaldehyde. The paraffin-embedded tissues were sectioned (5 *μ*m thickness) and stained with hematoxylin/eosin (H&E) or periodic acid-Schiff (PAS) to measure inflammatory cellular infiltration or mucus production, respectively. The quantitative analysis of peribronchial inflammation in H&E-stained lung slices was performed by determining the number of rings of inflammatory cells around the bronchi, and the quantification of mucus production was accomplished by assessing the number of PAS^+^ cells in the airway via the perimeter of the basement membrane (Pbm) as in our previous studies [25, 26].

### 2.4. Bronchoalveolar Lavage Fluid (BALF) and Cellular Analysis

BALF was collected by lavage of the left lung with 0.5 ml of ice-cold PBS. The fluid was centrifuged at 700 g at 4°C for 5 min. The cell pellets harvested from the BALF were resuspended in 200 *μ*l PBS. Total cells were counted using a hemocytometer, and differential cell counts were made by staining cytospins of BALF samples with Wright Stain solution (Sigma). At least 200 cells were counted for each mouse. The supernatants were stored at -80°C for ELISA analysis.

### 2.5. Enzyme-Linked Immunosorbent Assay (ELISA)

The production of IL-5, IL-4, and galectin-1 in BALF and anti-OVA IgE and IL-17 in plasma was measured by ELISA using specific kits (Cusabio, Wuhan, China) according to the protocols from the manufacturer. The specificities of IL-5, IL-4, IL-17, and galectin-1 were 7.8 pg/ml, 0.39 pg/ml, 11.75 pg/ml, and 0.078 ng/ml, respectively.

### 2.6. Immunohistochemistry

Gal-1 staining was performed in 5 *μ*m sections of paraffin-embedded lung tissue. Briefly, after being rehydrated in an alcohol gradient, the floating sections were rinsed and treated with 0.3% hydrogen peroxide and 0.5% Triton X-100 in PBS for 0.5 hours to quench endogenous peroxidase activity. Then, following a blocking step with 5% normal donkey serum (Vector Laboratories, Burlingame, CA) in PBST (PBS containing 0.5% Triton X-100) for one hour at 37°C, the sections were incubated with primary antibodies of mouse anti-galectin-1 (Santa Cruz Biotechnology, CA, USA) diluted in 1 : 100 in PBST overnight at 4°C. Amplification was performed with biotinylated goat anti-mouse IgG (1 : 200; Vector Laboratories) in PBST for 1 hour at 37°C. Positive staining was detected using a peroxidase-conjugated streptavidin complex, and color was developed using 0.05% 3,3′-diaminobenzidine (Sigma-Aldrich). The sections were mounted on glass slides in TB containing 0.5% gelatin and air-dried overnight before being dehydrated in ethanol-xylene, and coverslips were added using Permount. Photographs were taken using a Zeiss Axioskop 2 microscope (Carl Zeiss).

### 2.7. Quantitative Real-Time PCR

The total RNA from lung tissues was extracted using the trizol (Pufei) method and was evaluated using a One Drop OD-1000 spectrophotometer. Total RNA (1000 ng) was then reverse transcribed (TaKaRa) into cDNA, and mRNA levels of *β*-actin, eotaxin, EPX, galectin-1, IL-25, IL-33, and TSLP were analyzed via Q-PCR using a SYBR Green PCR Master Mix (TaKaRa) on a StepOne platform (Applied Biosystems). A Q-PCR system was applied in a 20 *μ*l volume for 40 cycles (15 s at 95°C and 1 min at 60°C). The gene expression levels were evaluated using the 2^−ΔΔCt^ method. The primers used are shown in supplementary Table S1.

### 2.8. Western Blotting

The lung tissues were homogenized using a homogenizer with RIPA buffer containing a protease inhibitor cocktail (Roche, Indianapolis, IN, USA) and phosphatase inhibitor PhosSTOP (Roche). Total protein was separated by SDS-PAGE and transferred to PVDF membranes (Millipore, Billerica, MA, USA). Membranes were blocked in 5% nonfat milk and then processed with primary antibodies: anti-p-ERK/ERK (1 : 1000), anti-p-JNK/JNK (1 : 1000), anti-p-P38/P38 (1 : 1000), anti-eotaxin (1 : 1000), and anti-IL-25 (1 : 1000) (Cell Signaling Technology Inc., Beverly, MA, USA); anti-galectin-1 (1 : 200) and anti-EPX (1 : 200) (Santa Cruz Biotechnology, CA, USA); and anti-GAPDH (1 : 5000) (KANGCHEN Biotech, Shanghai, China). All of the membranes were subsequently incubated with HRP-conjugated anti-rabbit IgG (Promega, Madison, WI, USA) and polyclonal rabbit anti-mouse IgG (Dako, Copenhagen, Denmark), and all of the blots were detected via enhanced chemiluminescence (ECL; Thermo Scientific). For quantitative analysis, the intensity of protein bands was determined using ImageJ 1.38x software (NIH, Bethesda, MD, USA).

### 2.9. Statistical Analysis

Statistical analysis was performed using SPSS 16.0. All of the data were expressed as the mean ± SD of triplicate samples and were representative of at least three separate experiments. Independent-sample *t*-tests or one-way ANOVA with a post hoc Bonferroni's test was used for all of the statistical analysis. Values of *P* < 0.05 were considered significant.

## 3. Results

### 3.1. rGal-1 Ameliorates Pathologic Features of Allergic Mice

**Figure 1:**
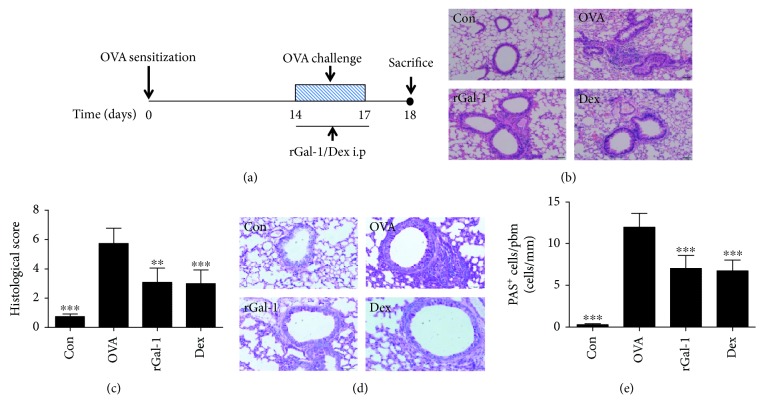
Treatment of rGal-1 ameliorates pathologic features of OVA-induced allergic mice. (a) Experimental protocol of establishment of allergic airway disease model in C57BL/6 mice. (b) Representative H&E staining of lung sections from C57BL/6 mice. Bars: 50 *μ*m. (c) Histological score of lung inflammation. (d) Representative periodic acid-Schiff (PAS) staining of lung sections from C57BL/6 mice. Bars: 50 *μ*m. (e) Quantification of PAS^+^ cells in the airway. Data were expressed as the means ± SD of 6 mice per group. Compared to the OVA group, ∗∗*P* < 0.01, ∗∗∗*P* < 0.001.

To examine the effects of rGal-1 in allergic airway inflammatory disease, C57BL/6 mice were sensitized and challenged with OVA as illustrated in Figure 1(a). Histological staining revealed that more inflammatory cells were recruited into the peribronchial regions in the lungs of the OVA group mice compared with the control group mice (Figures 1(b) and 1(c)). Concurrently, PAS^+^ cells surrounding the airway were significantly increased in the OVA group mice (Figures 1(d) and 1(e)). The OVA-challenged mice administered with rGal-1 or dexamethasone showed a significant reduction of inflammatory cell recruitment in the peribronchial regions (Figures 1(b) and 1(c)) as well as fewer PAS^+^ cells surrounding the airway (Figures 1(d) and 1(e)).

### 3.2. rGal-1 Reduces the Invasion of Inflammatory Cells and the Production of Inflammatory Cytokines in BALF

**Figure 2:**
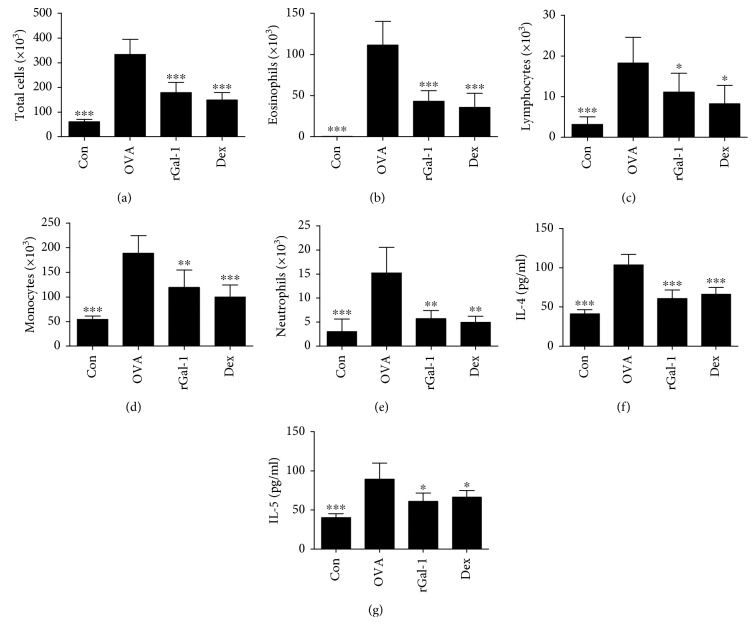
Treatment of rGal-1 reduces the invasion of inflammatory cells and production of inflammatory cytokines in BALF. (a–e) Total and differential cell counts in BALF. (f, g) Secretion levels of IL-4 and IL-5 in BALF. Data were expressed as the means ± SD of 6 mice per group. Compared to the OVA group, ∗*P* < 0.05, ∗∗*P* < 0.01, and ∗∗∗*P* < 0.001.

We counted the number of inflammatory cells and measured inflammatory cytokines in the BALF. The numbers of total cells, eosinophils, lymphocytes, monocytes, and neutrophils of the allergic mice were significantly elevated compared with those of the control mice (Figures 2(a)–2(e)). Moreover, the production of IL-5 and IL-4 in the BALF of the allergic mice was also significantly increased compared with that of the control mice (Figures 2(f) and 2(g)). Treatment with rGal-1 or Dex produced a significant decrease in released inflammatory cells, especially eosinophils (Figures 2(a)–2(e)). Concurrently, treatment with rGal-1or Dex significantly reduced the secretion of IL-5 and IL-4 in the BALF (Figures 2(f) and 2(g)).

### 3.3. Treatment of rGal-1 Decreases the Infiltration of Eosinophils and the Expression Levels of IL-25 in the Allergic Lung

**Figure 3:**
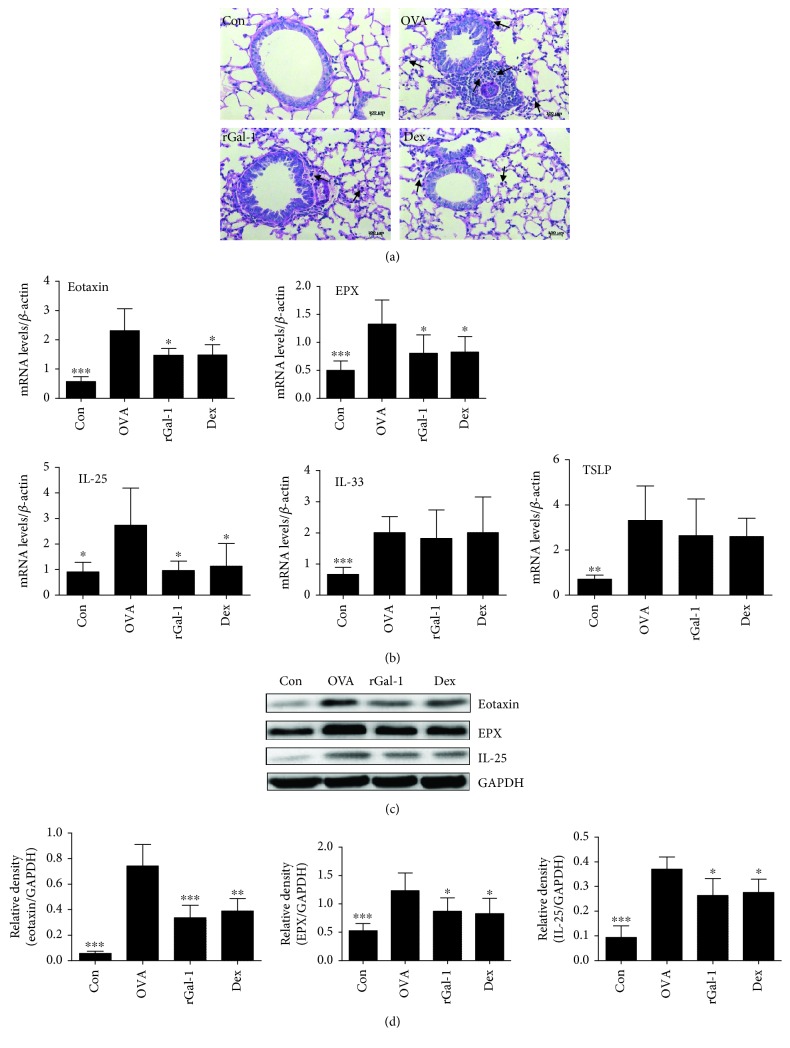
Treatment of rGal-1 decreases the infiltration of eosinophils and the expression levels of IL-25 in the allergic lung. (a) Representative periodic acid-Schiff (PAS) staining of lung sections and indication of eosinophils (arrowheads). Bars: 100 *μ*m. (b) Expressive mRNA levels of eotaxin, EPX, IL-25, IL-33, and TSLP in the lungs of mice by quantitative real-time PCR. (c) Measurement of protein expression of eotaxin, EPX, and IL-25 in the lungs of mice by Western blotting. (d) The relative level of these proteins in the lung. Data were expressed as the means ± SD of 6 mice per group. Compared to the OVA group, ∗*P* < 0.05, ∗∗*P* < 0.01, and ∗∗∗*P* < 0.001.

Histopathological analysis of the lungs from OVA-challenged mice showed increased infiltration of eosinophils compared to control mice (Figure 3(a)). Administration of rGal-1 or Dex resulted in a decrease in the infiltration of eosinophils (Figure 3(a)). These findings were further confirmed by measuring the decreased mRNA expression levels of the endogenous adhesion molecules eotaxin and EPX by quantitative real-time PCR in rGal-1 or Dex treated mice (Figure 3(b)). Next, we examined the protein expression levels of the two molecules by Western blotting. Similarly, the two proteins were significantly decreased by rGal-1 or Dex treatment (Figures 3(c) and 3(d)). To examine whether rGal-1 influenced epithelial cell function, we measured mRNA expression of IL-25, IL-33, and thymic stromal lymphopoietin (TSLP) in the lung by quantitative real-time PCR. The expression levels of each mRNA were significantly increased in the lungs of OVA-challenged mice (Figure 3(b)). However, only the mRNA expression levels of IL-25, neither IL-33 nor TSLP, were significantly decreased by treatment of rGal-1 or Dex relative to those of the untreated group (Figure 3(b)). Similarly, the protein expression levels of IL-25 were significantly reduced by rGal-1 or Dex treatment (Figures 3(c) and 3(d)).

### 3.4. rGal-1 Induces the Activation of the ERK Signaling Pathway in the Allergic Lungs

**Figure 4:**
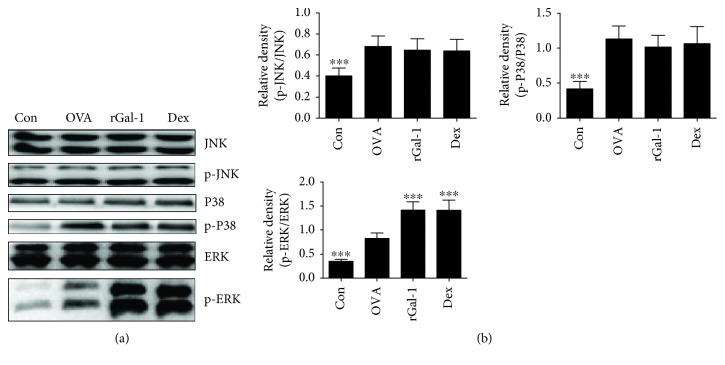
Treatment of rGal-1 induces the activation of the ERK signaling pathway in the allergic lung. (a) Measurement of MAPK protein expression in the lung of mice by Western blotting. (b) The relative level of MAPKs in the lung. Data were expressed as the means ± SD of 6 mice per group. Compared to the OVA group, ∗∗∗*P* < 0.001.

To identify the downstream molecular signaling pathways mediated by rGal-1 treatment in OVA-induced allergic airway inflammation, we analyzed the phosphorylation levels of mitogen-activated protein kinases (MAPKs) in the lungs by Western blotting. OVA group mice exhibited significantly increased protein phosphorylation of MAPKs compared to control mice (Figures 4(a) and 4(b)). However, only the expression levels of p-ERK, neither p-JNK nor p-P38, were significantly increased by treatment with rGal-1 or Dex relative to that in the OVA group (Figures 4(a) and 4(b)).

### 3.5. The Expression of Gal-1 Is Induced in the Allergic Lungs

**Figure 5:**
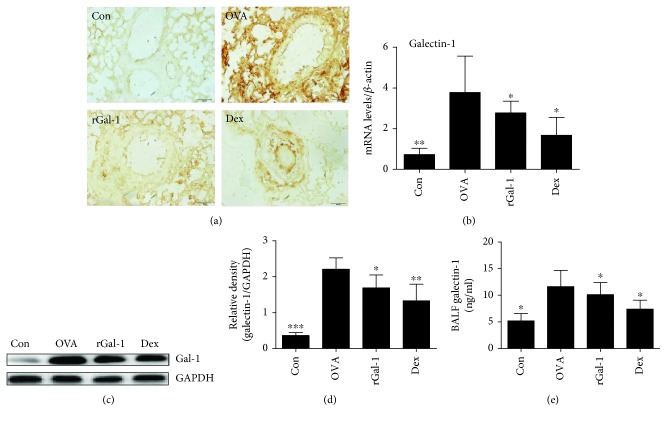
Expression levels of Gal-1 in the allergic lungs. (a) Representative Gal-1 expression of lung sections by immunohistochemistry from C57BL/6 mice. Bars: 50 *μ*m. (b) Expressive mRNA levels of Gal-1 in the lung of mice by quantitative real-time PCR. (c) Measurement of Gal-1 protein expression in mice by Western blotting. (d) The relative protein levels of Gal-1 in the lung. (e) Production levels of Gal-1 in BALF by ELISA. Data were expressed as the means ± SD of 6 mice per group. Compared to the OVA group, ∗*P* < 0.05, ∗∗*P* < 0.01, and ∗∗∗*P* < 0.001.

We measured Gal-1 expression in the lungs of the mice. Gal-1 expression levels in the lungs of OVA-challenged mice were elevated compared to those of control mice by immunohistochemistry analysis (Figure 5(a)). Furthermore, the mRNA and protein expression levels of Gal-1 in the lungs were significantly increased in OVA-challenged mice compared to the control mice by quantitative real-time PCR and Western blotting analysis, respectively (Figures 5(b)–5(d)). Similarly, the production of soluble Gal-1 was also significantly higher in the BALF of allergic mice compared to that of control mice by ELISA analysis (Figure 5(e)). Most importantly, the expression of Gal-1 was significantly decreased in the lungs of OVA-challenged allergic mice by treatment with rGal-1 or Dex (Figure 5(a)). The similar effects were also supported by measurement of Gal-1 mRNA and protein levels in the lungs and soluble Gal-1 levels in the BALF (Figures 5(b)–5(e)).

### 3.6. rGal-1 Reduces the Plasma Levels of IL-17 and Anti-OVA IgE in Allergic Mice

**Figure 6:**
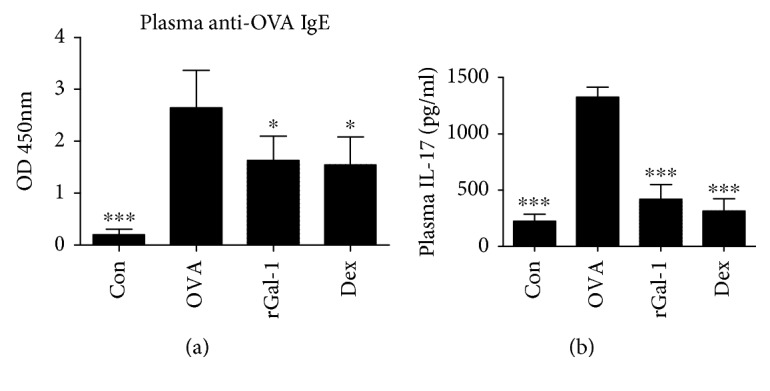
Treatment of rGal-1 reduces the plasma levels of anti-OVA IgE and IL-17 in allergic mice. (a) Plasma levels of anti-OVA IgE by ELISA. (b) Plasma levels of IL-17 by ELISA. Data were expressed as the means ± SD of 6 mice per group. Compared to the OVA group, ∗*P* < 0.05, ∗∗∗*P* < 0.001.

We assessed the systemic immune response by measuring plasma anti-OVA IgE and IL-17 levels using ELISA. The plasma IgE and IL-17 levels of the OVA group mice were significantly higher than those of the control group mice (Figures 6(a) and 6(b)). Moreover, both rGal-1 and Dex treatments markedly decreased the plasma levels of OVA-induced IgE and IL-17 relative to the OVA group mice (Figures 6(a) and 6(b)).

## 4. Discussion

In the present study, we assessed the effects of pharmacological treatment with rGal-1 on an OVA-induced murine model of allergic airway inflammation. Applying histological, biochemical, and molecular analyses, we showed that systemic rGal-1 treatment was as effective as dexamethasone (Dex) in the inhibition of the allergic airway inflammation. These local immunomodulatory effects were correlated with the activation of extracellular signal-regulated kinase (ERK) signaling pathway and downregulation of endogenous Gal-1 expression. Moreover, both rGal-1 and Dex reduced the plasma concentrations of anti-OVA immunoglobulin E (IgE) and IL-17. The present results revealed that rGal-1, as well as Dex, is an effective treatment for allergic airway inflammation in a murine model and suggested that Gal-1 may be a potential pharmacological target for allergic airway inflammatory diseases.

The anti-inflammatory effects of exogenous Gal-1 have been confirmed in various disease models [27–29]. Furthermore, using Gal-1 as a therapeutic agent has been examined in various models of allergic inflammation apart from asthma [11–13]. Treatment with rGal-1 in mice with IgE-mediated allergic conjunctivitis or OVA-induced atopic dermatitis resulted in resolution of clinical signs and reduced levels of Th2 cytokines and chemokines [11, 13]. Furthermore, in a model of oral-intestinal allergy syndrome, challenge of mice sensitized to peanut extracts along with the administration of Gal-1 decreased intestinal allergic inflammation relative to mice sensitized to peanut extracts alone by restoring IL-10 expression in the intestine [12]. Although there are no previous studies of Gal-1 as a therapeutic agent for allergic airway inflammation, allergen-challenged Lgals^−/−^ mice have shown increased airway inflammatory cell infiltration and proinflammatory cytokine secretion [23]. In accordance with these findings, we found that OVA-induced allergic inflammation, including accumulation of leukocytes in BALF, inflammatory cell infiltration into the peribronchial area, goblet cell hyperplasia in the airways, and proinflammatory cytokine production by T cells, such as IL-4 and IL-5, were significantly decreased by rGal-1 or Dex treatment. These results suggest that Gal-1 may be involved in the regulation of Th2 response in allergic airway inflammation.

To further characterize the role of rGal-1 in allergic airway inflammation, we analyzed cell infiltration in the lungs of OVA-induced mice. Gal-1 suppressed neutrophil extravasation and mast cell degranulation at sites of inflammation [15, 16, 30]. In addition, low concentrations of Gal-1 promoted eosinophil adhesion, but high concentrations of Gal-1 resulted in eosinophil apoptosis [23]. Furthermore, rGal-1 inhibited eosinophil migration in OVA-challenged atopic dermatitis mice [13]. In line with these results, we observed similar inhibitory effects on allergic airway inflammation with rGal-1 treatment, including the inhibition of eosinophil influx into the allergic airways. Moreover, we also found that mRNA and protein expression levels of eotaxin and EPX were significantly decreased in rGal-1-treated mice compared to those in untreated mice, suggesting that the exogenous action of Gal-1 was essential to mediate the activation of eosinophils in OVA-induced mice. Studies have shown that epithelial-derived cytokines, including IL-33, IL-25, and thymic stromal lymphopoietin (TSLP), regulate type 2 immune response to aeroallergen exposure in the lungs [31]. Thus, we also measured the mRNA and protein expression levels of these cytokines in the lungs. However, only the expression levels of IL-25, neither IL-33 nor TSLP, were significantly decreased in the lung by rGal-1 treatment. This novel finding suggests that Gal-1 may partly regulate type 2 immune responses by affecting epithelial cell function.

Higher expression of endogenous Gal-1 was observed in the lungs of OVA-challenged mice relative to control mice, which is similar to a previous study [23]. Moreover, Gal-1 expression levels were also significantly higher in OVA-induced atopic dermatitis and allergic conjunctivitis mice than in control mice [11, 13]. On the one hand, rGal-1 markedly increased Gal-1 levels and provoked eosinophilia in OVA-induced allergic conjunctivitis mice compared to control mice at 4-hour point [11]. On the other hand, rGal-1 decreased Gal-1 levels and inhibited eosinophilia in OVA-induced atopic dermatitis mice relative to control mice after 24 hours [13]. Additionally, OVA-challenged Gal-1-deficient mice exhibited increased recruitment of eosinophils in the airway compared to corresponding wild-type mice [23]. The present study also found that rGal-1 decreased Gal-1 expression and inhibited the infiltration of eosinophils in the airway after 24 hours. These findings may suggest that Gal-1 expression in the allergic lung is time- and eosinophil-dependent.

Mitogen-activated protein kinases (MAPKs) are well-conserved signaling pathways that include three subtypes: c-jun N-terminal kinases (JNK), extracellular signal-regulated kinases (ERK), and P38 proteins, which are critical to activating the expression of multiple genes that mediate the immune response [32]. Moreover, MAPKs have been proposed to be involved in the pathogenesis of asthma, and the phosphorylation levels of MAPKs are upregulated in animal models of asthma [33]. In line with these previous studies, we found that the expression levels of p-MAPKs were significantly increased in allergic mice relative to those in control mice, suggesting that the MAPK signaling pathways are activated in OVA-challenged allergic mice. In the absence of additional inflammatory stimuli or tissue injury, Gal-1 contributed to neutrophil migration via the activation of P38 [34]. At low concentrations, Gal-1 promoted eosinophil adhesion and inhibited ERK activation. However, exposure to higher concentrations of this lectin resulted in ERK-dependent eosinophil apoptosis [23]. Increased phosphorylation levels of ERK were observed in OVA-induced allergic conjunctivitis [11], atopic dermatitis [13], and allergic asthma mice [23]. Moreover, treatment with rGal-1 in OVA-induced allergic conjunctivitis [11] and atopic dermatitis mice [13] contributed to the activation of p-ERK. In accordance with these previous reports, we herein found that only the expression levels of p-ERK, neither p-JNK nor p-P38, were significantly increased relative to untreated mice. These results suggest that Gal-1 can mediate allergic airway inflammation through the activation of the ERK signaling pathway in OVA-induced allergic airway inflammatory mice.

Administration of rGal-1, as well as Dex, significantly reduced anti-OVA IgE compared to the untreated OVA group. The similar effects of rGal-1 in mice with OVA-induced allergic conjunctivitis were observed at four hours after the final OVA challenge, but there was no difference 24 hours after the final OVA challenge [11]. In addition, rGal-1 did not reduce plasma IgE levels 24 hours after the last OVA challenge in an OVA-induced atopic dermatitis mouse model [13]. However, another line of research related to long-term treatment with rGal-1 and allergen-specific immunotherapy found that rGal-1 markedly decreased the plasma IgE levels in an OVA-induced intestinal allergy mouse model [35]. These differences may suggest that downregulation of IgE by rGal-1 in allergic inflammation was time-dependent. We also observed that the increased plasma levels of IL-17 were significantly decreased by rGal-1 and Dex treatment in OVA-induced allergic airway mice, and a similar effect was observed in OVA-induced atopic dermatitis mice [13]. These findings suggest that Gal-1 may play a crucial role in regulation of systemic allergic inflammation.

## 5. Conclusion

Overall, the present findings demonstrate that systemic rGal-1 treatment was as effective as dexamethasone (Dex) in the reduction of allergic airway inflammation through activation of the ERK signaling pathway in acute asthma mice. Therefore, the Gal-1 pathway may provide novel therapeutic approaches for allergic airway diseases.

## Data Availability

The data used to support the findings of this study are included either within this article or within the supplementary information files.

## Supplementary Materials

Supplementary Materials Table S1: qRT-PCR primers of genes.
