# Supplementary material for: Anti-inflammatory Property of Galectin-1 in a Murine Model of Allergic Airway Inflammation
Source: J Immunol Res. 2019 May 12;2019:9705327. doi: 10.1155/2019/9705327 (PMC6535876; doi:10.1155/2019/9705327)
Supplement: Supplementary Materials — Table S1: qRT-PCR primers of genes. [file 9705327.f1.docx]

**Supplementary Materials**

Table S1. The detail information of primers

| Primers | Sequence (5’to 3’) |
| --- | --- |
| β-Actin | Forward :ACTCCTATGTGGGTGACGAG |
|  | Reverse :CATCTTTTCACGGTTGGCCTTAG |
| eotaxin | Forward :TGCTCACGGTCACTTCCTTC |
|  | Reverse :CTTGAAGACTATGGCTTTCAGGGTG |
| EPX | Forward :CACGGCCTTCCAGGATACAA |
|  | Reverse :GATGTCAATGTTGTCGGGCG |
| Galectin-1 | Forward :CGGGTGGAGTCTTCTGACTG |
|  | Reverse: GCACAAAGCTCTTGGCGTC |
| IL-25 | Forward :CGGCATGTACCAGGCTGTTG |
|  | Reverse :CTCCACTTCAGCCACTCCTC |
| IL-33 | Forward :GGCTCACTGCAGGAAAGTACA |
|  | Reverse :TTGGTCTTCTGTTGGGATCTTCT |
| TSLP | Forward: GGACTGTGAGAGCAAGCCAG |
|  | Reverse: TCCGGGCAAATGTTTTGTCG |
